# Reliability of Routinely Collected Hospital Data for Child Maltreatment Surveillance

**DOI:** 10.1186/1471-2458-11-8

**Published:** 2011-01-05

**Authors:** Kirsten McKenzie, Debbie A Scott, Garry S Waller, Margaret Campbell

**Affiliations:** 1 National Centre for Health Information Research and Training, School of Public Health, Queensland University of Technology, Victoria Park Road, Kelvin Grove QLD Australia 4059

## Abstract

**Background:**

Internationally, research on child maltreatment-related injuries has been hampered by a lack of available routinely collected health data to identify cases, examine causes, identify risk factors and explore health outcomes. Routinely collected hospital separation data coded using the International Classification of Diseases and Related Health Problems (ICD) system provide an internationally standardised data source for classifying and aggregating diseases, injuries, causes of injuries and related health conditions for statistical purposes. However, there has been limited research to examine the reliability of these data for child maltreatment surveillance purposes. This study examined the reliability of coding of child maltreatment in Queensland, Australia.

**Methods:**

A retrospective medical record review and recoding methodology was used to assess the reliability of coding of child maltreatment. A stratified sample of hospitals across Queensland was selected for this study, and a stratified random sample of cases was selected from within those hospitals.

**Results:**

In 3.6% of cases the coders disagreed on whether any maltreatment code could be assigned (definite or possible) versus no maltreatment being assigned (unintentional injury), giving a sensitivity of 0.982 and specificity of 0.948. The review of these cases where discrepancies existed revealed that all cases had some indications of risk documented in the records. 15.5% of cases originally assigned a definite or possible maltreatment code, were recoded to a more or less definite strata. In terms of the number and type of maltreatment codes assigned, the auditor assigned a greater number of maltreatment types based on the medical documentation than the original coder assigned (22% of the auditor coded cases had more than one maltreatment type assigned compared to only 6% of the original coded data). The maltreatment types which were the most 'under-coded' by the original coder were psychological abuse and neglect. Cases coded with a sexual abuse code showed the highest level of reliability.

**Conclusion:**

Given the increasing international attention being given to improving the uniformity of reporting of child-maltreatment related injuries and the emphasis on the better utilisation of routinely collected health data, this study provides an estimate of the reliability of maltreatment-specific ICD-10-AM codes assigned in an inpatient setting.

## Background

Child maltreatment is a major public health problem worldwide. A 2005 report estimated the prevalence of child maltreatment in Australia as affecting 10-20% of children [1]. Research has been hampered by poorly validated statistics with the World Health Organization (WHO) stating that a lack of data is a hindrance to understanding the magnitude and consequences of child maltreatment [2]. The WHO has recommended uniform reporting of child maltreatment-related injuries and deaths [3], with an increased emphasis internationally on the importance of health professionals in identifying and documenting suspected child maltreatment in medical records [3,4]. The operational definition of child maltreatment, according to the WHO is:

"All forms of physical and/or emotional ill-treatment, sexual abuse, neglect or negligent treatment or commercial or other exploitation, resulting in actual or potential harm to the child's health, survival, development or dignity in the context of a relationship of responsibility, trust or power" p. 59 [2].

Hospital separation data are a key source of standardised data which are routinely used to assess population health, with these data coded using the International Statistical Classification of Diseases and Related Health Problems (ICD) developed by WHO [5]. In many countries, clinical modifications are made to the ICD to include greater specificity for reporting. In Australia as well as several other countries (New Zealand, Ireland, Germany, Romania, Slovenia and Saudi Arabia [6]), hospital separation data are coded using the Australian clinical modification, ICD-10-AM [7].

Hospital separation data rely on detailed documentation in hospital records by health professionals. Clinical coders in the hospital setting are responsible for reviewing medical records and abstracting the necessary information to assign ICD-10-AM codes to reflect the patient's episode of care. If there is a lack of adequate documentation in the record, morbidity coders are limited in the range of codes they can accurately assign. Zeigler et al found that in Emergency Department (ED) records, only 20% of cases of fractures in children under three years of age had documentation to indicate whether child maltreatment had been considered by the emergency physician, and 27% of cases had inadequate documentation to assess the consistency of the injury with the case history [8]. Limbos et al found that, of those cases who had been discharged with a diagnosis of child maltreatment, only 45% of medical records had documentation specifying the history of injuries and over a quarter of cases omitted a description of the physical examination findings [9]. The medical record is a key to information sharing and communication among clinical staff and is also the primary source of documentation for routine clinical coding. Therefore, limited clinical documentation around the suspicion of child maltreatment may hamper the process of identification and coding of these cases.

Clinical coders undergo significant training and know and understand the stringent requirements involved in accurately assigning ICD-10_AM codes to medical records. In order for a definitive maltreatment code to be assigned the coder must find clear evidence and a definitive diagnosis of maltreatment in the medical record[10]. If the record indicates an injury is 'queried' or 'suspicious' but there are no definitive statements about maltreatment, a range of possible codes may be assigned, and the authors have described these ICD-10-AM codes in McKenzie et al (2010) [11].

There has been no research which examines the reliability of coding of child maltreatment in hospital separation data in Australia and limited research internationally. The aim of this study was to assess the reliability of coding of child maltreatment in hospital discharge data in Queensland.

## Methods

A retrospective medical record and coding review was used to assess the reliability of coding of child maltreatment in hospital records in Queensland, Australia. The sampling process was conducted in two phases. Initially, a stratified sample of hospitals across Queensland was conducted and then a stratified random sample of cases from within each selected hospital was conducted.

### Hospital Sample

The aim of hospital selection was to identify a range of large, medium and small caseload public hospitals from metropolitan, rural and remote areas throughout Queensland. Eligible hospitals for inclusion in the sampling process were those that: were categorised as a public hospital, had an accident and emergency department and an acute care service, treated paediatric patients, and had more than 1000 admissions per year (53 of 99 Queensland public hospitals with an emergency department satisfied all criteria). It was estimated that approximately 20 hospitals across Queensland could be sampled based on the resources available (budget, time, staff). Within this sample, the aim was to collect a sample of patient records from an equal number of large (>= 30,000 admissions/year), medium (10,000-29,999 admissions/year), and small (<10,000 admissions/year) hospitals, and the final sample included 7 large hospitals, 7 medium hospitals and 6 small hospitals. from large and medium hospitals and 6 from small hospital. Once ethical approvals were obtained, patient sampling was undertaken.

### Patient Sample

Patient sample selection was conducted using the Queensland Health Admitted Patient Data Collection (QHAPDC) [12]. This database contains ICD-10-AM coded data for every hospital discharge occurring from any public hospital, licensed private hospital and day surgery unit since 1985 in Queensland. The admission year range for case selection was 2003 to 2006. The age range for case selection was under 18 years to comply with the operational definition of child in the *Child Protection Act *(1999) which governs Queensland child protection [13].

The coding of child-maltreatment had been found to be rare (0.3% of hospitalisations in Australia in 2005/06 for children had any maltreatment code assigned) [11]. To ensure a sufficient sample of cases of child maltreatment could be reviewed, cases were first stratified into three groups based on the presence of the following codes as either a principal diagnosis or an additional diagnosis: definitive maltreatment codes, possible maltreatment codes, and injury codes with an external cause of unintentional intentSpecific details of these code groupings are as follows:

• Strata 1 (Definitive maltreatment code) - This strata included all cases with a definitive maltreatment code (T74 Maltreatment Syndrome) assigned as either a principal diagnosis or an additional diagnosis in the patient's hospital separation data.

• Strata 2 (Possible maltreatment code) - This strata included all cases that weren't grouped into Strata 1, but had any of a range of possible maltreatment codes [11], including: (a) one or more of the ICD-10-AM codes Z04.4 Examination and observation following alleged rape and seduction, Z04.5 Examination and observation following other inflicted injury, Z61.4 Problems related to alleged sexual abuse of child by person within primary support group, Z61.5 Problems related to alleged sexual abuse of child by person outside primary support group, Z61.6 Problems related to alleged physical abuse of child, Z62.0 Inadequate parental supervision and control, Z62.3 Hostility towards and scapegoating of child, Z62.4 Emotional neglect of child, Z62.5 Other problems related to neglect in upbringing, Z62.6 Inappropriate parental pressure and other abnormal qualities of upbringing reported as a principal or additional diagnosis, (b) an ICD-10-AM external cause code in the range X85-Y09 Assault codes, where the perpetrator was identified at the fifth-digit with a value of 1 Parent, 2 Other family member, or 3 Carer for 15 to 17 year old children or a fifth-digit with a value of 1 Parent, 2 Other family member, 3 Carer, 8 Other specified person, or 9 Unspecified person for patients under 15 years of age, or (c) an ACHI procedure code of 5830600 Radiography of the whole skeleton or 9608400 Physical abuse/violence/assault counselling reported in any of the procedure codes assigned.

• Strata 3 (Unintentional injury code) - This strata included all cases admitted to hospital with an injury diagnosis (ICD-10-AM code range S00-T98) reported as a principal or additional diagnosis with an external cause in the unintentional cause code range (V00-X59) who weren't in Strata 1 or Strata 2.

The sample size for patient records was determined by a number of factors, including budget, resources and statistical power considerations. A target of 1500 inpatient records was set initially, with a target of 500 records per strata. To take into account the different caseloads of different hospitals and to approximate a probability-based result, the number of cases for selection at each hospital was stratified according to the size of the hospital. For Large Hospitals which were Principal Referral/Specialised Children's Hospitals a maximum of 40 cases per strata was set, for other Large Hospitals a maximum of 20 cases per strata was set, and for Medium/Small hospitals a maximum of 10 cases per strata were set.

While 500 records per strata was the initial target, preliminary screening of the QHAPDC data found that Strata 1 only contained 218 eligible cases and Strata 2 only contained 293 eligible cases, resulting in smaller numbers of cases than expected available for review in these two Strata.

### Data collection

Details on the age range, range of admission dates and specific ICD-10_AM codes to include in the sample (as described above) were provided to the State Health department, and the department extracted a random sample of unit record numbers (URN) and hospital identifiers. A list of URNs was provided to Health Information Managers within each hospital, and they extracted the hard copy medical record for review on-site. An ACCESS database was specifically designed by KM to facilitate the systematic collection of data across researchers and hospitals. Training for the data collection was based on a detailed training manual and all data collectors and coders were provided with this training and a copy of the manual for reference as necessary during the data collection phase. The database was piloted prior to implementation to ensure that all researchers and coders were collecting the same information and recording it in the same format across all sites. An expert coding auditor and a researcher visited each site to review the medical records. The collection methods and database design ensured that the coding auditor was blinded to the all existing codes (as assigned on patient discharge) during the data abstraction and recoding processes. The coding auditor used the database to review the records to obtain information relevant for assigning ICD-10-AM codes, allocated the appropriate codes, and then compared these to the codes assigned by the original coder.,

The medical record review was contingent on the availability of the medical record during the time that the researchers and coding auditors were on-site. If the patient record was required by another hospital department, for example if the patient had an outpatient appointment or had been readmitted for some reason or the record was required by the legal department, at the time of the data abstraction and it didn't become available during that time, the record was missed.

### Data analysis

Kappa statistics were used to examine the interrater reliability for strata assignment. Also, ICD-10-AM maltreatment codes were grouped to create variables to flag the presence of each of the main forms of maltreatment (neglect, physical abuse, sexual abuse, psychological abuse, and other/unspecified abuse) as coded by the original coder and the auditor (For more detail on these code groupings see McKenzie et al 2010 [11]. Analysis for this study was focused on assessing the documentation and coding of each admission therefore, there was no need to account for multiple admissions for a child. Sensitivity and specificity were calculated comparing the two coders' assignment of cases to maltreatment strata and the two coders' assignment of maltreatment types.

## Results

### Sample Characteristics

Between 80% and 100% of cases in the target sample were available to be reviewed onsite, with an average review rate of 92.2% of target records. By strata, 94.0% of the cases in Strata 1, 86.3% of the cases in Strata 2, and 94.9% of the cases in Strata 3 were reviewed from the target sample. Of the total population of hospitalised children in Queensland between 2003 and 2006 with a definite maltreatment code assigned, 82.3% of cases were reviewed, 33.9% with a possible maltreatment code, and 1.0% with an unintentional injury code.

Table 1 shows age and sex distributions and for these calculations, only primary cases (i.e. the first separation for each child) were included (884 children in the sample of 923 separations). There were differences in the age and sex distribution by maltreatment strata, with a larger number of females than males in the two maltreatment strata. Almost 60% of the sample with a definitive code and 68% of the sample with a possible code were female, while females comprised only 34% of the unintentional injury sample. Age distributions varied in each strata with the largest proportion of children with a definitive code aged <5 years (over 70%), while the largest proportion of children with a possible code were aged >10 years (over 70%). Almost half of the males in the definitive strata were aged <1 year, compared to only a quarter of the females. In contrast, 38% of females in the in the definitive strata were aged >6 years, compared to only 15% of males.

**Table 1:** Age Groups and Sex Distribution of Children by Maltreatment Strata

Sex and Age Groups	Definitive Code		Possible Code		Unintentional Injury Code		Total	
	n	Col%	n	Col%	n	Col%	n	Col%
Males								
<1	39	48.1	8	11.1	9	3.0	56	12.2
1-5	30	37.0	16	22.2	69	22.6	115	25.1
6-9	4	4.9	13	18.1	57	18.7	74	16.2
10-14	7	8.6	27	37.5	93	30.5	127	27.7
15-17	1	1.2	8	11.1	77	25.2	86	18.8
Total	81	100	72	100	305	100	458	100

Females								
< 1	31	26.1	8	5.3	9	5.8	48	11.3
1-5	43	36.1	28	18.5	51	32.7	122	28.6
6-9	15	12.6	12	7.9	39	25.0	66	15.5
10-14	22	18.5	57	37.7	29	18.6	108	25.4
15-17	8	6.7	46	30.5	28	17.9	82	19.2
Total	119	100	151	100	156	100	426	100

Total								
< 1	70	35.0	16	7.2	18	3.9	104	11.8
1-5	73	36.5	44	19.7	120	26.0	237	26.8
6-9	19	9.5	25	11.2	96	20.8	140	15.8
10-14	29	14.5	84	37.7	122	26.5	235	26.6
15-17	9	4.5	54	24.2	105	22.8	168	19.0

Total	200	100	223	100	461	100	884	100

### Strata Comparison

Original strata assignment was compared to the recoded strata assignment to examine the concordance of assigned maltreatment strata (See Table 2). The coders agreed on the assignment of definitive maltreatment codes for 77.4% of cases assigned to this stratum by the auditor, agreed on the assignment of possible maltreatment codes for 87.4%, and agreed on the assignment of unintentional injury codes for 94.8% of cases. The interrater reliability of coders for assignment of cases into the three maltreatment strata was 0.818 (Cohen's kappa).

**Table 2:** Comparison of Maltreatment Strata Assigned by Original Coder and Auditor

	New Intent							
**Original Intent**	**Definitive Code**		**Possible Code**		**Unintentional injury**		**Total**	
	**n**	Col%	**n**	Col%	**n**	Col%	**n**	Col%

Definitive Code	182	77.4	19	9.2	4	0.8	205	22.2
Possible Code	52	22.1	181	87.4	21	4.4	254	27.5
Unintentional injury	1	0.4	7	3.4	456	94.8	464	50.3

Total	235	100	207	100	481	100	923	100

The sensitivity and specificity were calculated comparing those cases where maltreatment was coded (combining definitive and possible maltreatment codes) and those cases where unintentional injury was coded (See Table 3). The sensitivity (i.e. of coding maltreatment when maltreatment was documented) was 0.982 and the specificity (i.e. of not coding maltreatment when maltreatment wasn't documented) was 0.948.

**Table 3:** Sensitivity and Specificity of Abuse Group Assignment

	New Intent					
**Original Intent**	**Definitive or Possible Code**		**Unintentional Injury Code**		**Total**	
	**n**	**Col%**	**n**	**Col%**	**n**	**Col%**

Definitive or Possible Code	434	98.2	25	5.2	459	49.7
Unintentional Injury Code	8	1.8	456	94.8	464	50.3

Total	442	100	481	100	923	100

There were 33 cases out of the 923 (3.6% of cases) reviewed where there was disagreement in whether any maltreatment code could be assigned (i.e. where the original coder assigned a definite/possible maltreatment code and the auditor assigned an unintentional injury code OR where the auditor assigned definite/possible maltreatment code and the original code assigned an unintentional injury code). Of these 33 cases, there were 5 cases where there was disagreement about definite maltreatment versus unintentional injury. All provided documentation of possible maltreatment in the records,, though there were no definitive statements that the injury being treated was a direct result of maltreatment. In almost all of the 28 cases where the disagreement was related to possible versus unintentional injury, the main difference was whether or not a maltreatment code to identify a history of maltreatment (i.e. as per the Z codes described in the method section) was assigned.

### Type Comparison

For cases where *any *maltreatment code was assigned by *either *coder, the number of maltreatment types assigned by each coder was compared (See Table 4). The original coder assigned only one maltreatment type for over 90% of cases, two maltreatment types for around 5% of cases, and three maltreatment types for less than 1% of cases. The auditor assigned only one maltreatment type for 72% of cases, two maltreatment types for around 17% of cases, and three maltreatment types for 5% of cases.

**Table 4:** Comparison of Number of Maltreatment Type Assigned by Original Coder and Auditor

	Auditor Number of Maltreatment Types									
**Original no. Maltreatment Types**	**No Types**		**One Type**		**Two Types**		**Three Types**		**Total**	
	**n**	**Col%**	**n**	**Col%**	**n**	**Col%**	**n**	**Col%**	**n**	**Col%**

No Types	2	8.0	11	3.3	1	1.2	0	0.0	14	3.0
One Type	22	88.0	322	95.3	62	77.5	19	79.2	425	91.0
Two Types	0	0.0	5	1.5	15	18.8	5	20.8	25	5.4
Three Types	1	4.0	0	0.0	2	2.5	0	0.0	3	0.6

Total	25	100	338	100	80	100	24	100	467	100

The original maltreatment type assignment was compared to the recoded maltreatment type assignment to examine the concordance of assigned maltreatment types (neglect, physical, sexual, psychological, and other) (See Table 5). For Auditor Maltreatment Type, the column headings 'Yes' and 'No' refer to the maltreatment types signified in the row heading. The sensitivity and specificity were calculated for each maltreatment type (using the auditor assigned types as the gold standard). For neglect, the sensitivity was 61.4% and the specificity was 94.7%. For physical abuse, the sensitivity was 72.6% and the specificity was 90.8%. For sexual abuse, the sensitivity was 86.5% and the specificity was 93.6%. For psychological abuse, the sensitivity was 8% and the specificity was 98.6%. For other or unspecified abuse, the sensitivity was 62.5%and the specificity was 87.3%.

**Table 5:** Comparison of Maltreatment Type Assigned by Original Coder and Auditor

	Auditor Maltreatment Type				
**Original Maltreatment Type**	**Yes**		**No**		**Total**
	**n**	**Col%**	**n**	**Col%**	**n**

Neglect					
Yes	113	61.4	15	5.3	128
No	71	38.6	268	94.7	339
Total	184	100	283	100	467

Physical					
Yes	127	72.6	27	9.2	154
No	48	27.4	265	90.8	313
Total	175	100	292	100	467

Sexual					
Yes	147	86.5	19	6.4	166
No	23	13.5	278	93.6	301
Total	170	100	297	100	467

Psychological					
Yes	2	8.0	6	1.4	8
No	23	92.0	436	98.6	459
Total	25	100	442	100	467

Other					
Yes	70	62.5	45	12.7	115
No	42	37.5	310	87.3	352
Total	112	100	355	100	467

## Discussion

This study examined the reliability of coding of child maltreatment in hospital separation data, finding an interrater reliability of 0.818 for the assignment of cases to strata. For only 3.6% of cases overall, the coders disagreed on whether *any *maltreatment code could be assigned (definite or possible maltreatment code compared to an unintentional injury code), with the auditor assigning an unintentional injury code to 5% of cases originally coded with a maltreatment code and assigning a maltreatment code to less than 2% of cases originally coded as unintentional. The review of these cases where discrepancies existed revealed that all cases had some indications of risk documented in the records, though the documentation was unclear regarding whether maltreatment was evident, hence affecting the certainty of coding. Thus researchers can be reasonably confident in the specificity of coded data for child maltreatment. Given the strict coding rules around the assignment of maltreatment codes, which rely on clear documentation to support code assignment, selection of cases based on the presence of maltreatment codes is highly likely to provide a sample of cases with documentation of maltreatment in their medical records. While documentation of maltreatment in the medical records is not a definitive indicator of actual maltreatment, these cases are arguably a high risk group for further investigation.

For 7.7% of cases overall, the main source of disagreement between coders was whether a definitive maltreatment code could be assigned versus a possible maltreatment code. While this only represented 7.7% of cases overall, it represented 15.5% of cases originally assigned as definite or possible maltreatment that were recoded to a more or less definite strata. Potential flags in the ICD for maltreatment-related events included both codes that signified a current episode of maltreatment and codes which signified a prior history of maltreatment. For a clinical coder to apply a definitive maltreatment code there must be clear clinical documentation of evidence of maltreatment. If documentation in the medical record indicates that the cause of the injury/disease is 'queried' or 'suspicious' of maltreatment, but evidence of further investigation to substantiate it is not documented, the coder cannot assign a definitive maltreatment code. Instead the coder may assign a range of codes indicating possible maltreatment (such as Z04.4 Examination and observation following alleged rape and seduction) or problems related to previous alleged maltreatment (Z61.4 Problems related to alleged sexual abuse of child by person within primary support group) [14]. The assignment of any maltreatment codes (current or prior) as a co-morbidity in Australian hospital separation data the patient must have been treated for and/or had their hospital stay extended due to the condition which was coded. Hence, researchers wanting to use maltreatment codes to identify cases where the presentation directly relates to a definite case of current maltreatment, as opposed to a possible case of current maltreatment or a history of previous maltreatment, would need to do further investigation at the medical record level to rule cases out or in.

Furthermore, in terms of the number and type of maltreatment codes assigned, this study found that the auditor assigned a greater number of maltreatment types based on the medical documentation than the original coder assigned (22% of the auditor coded cases had more than one maltreatment type assigned compared to only 6% of the original coded data). The maltreatment types which were the most 'under-coded' by the original coder were psychological abuse (only 2 cases out of the 25 cases of psychological abuse assigned by the auditor were coded by the original coder), and neglect (only 113 cases out of the 184 cases of neglect assigned by the auditor were coded by the original coder). Cases coded with a sexual abuse code showed the highest level of reliability. This has implications for future research, given the underestimates that would be likely if cases were sampled by specific maltreatment type alone. Researchers with a specific interest in selecting specific types of maltreatment from coded health data sets may need to select a broad sample of cases with any maltreatment code assigned initially, and then conduct a more in-depth medical record review (such as conducted in this study) to ensure more complete case capture.

This study found that selection of cases coded with an unintentional injury cause found only a small number of cases where further review resulted in a maltreatment code assignment (less than 2% of the cases originally coded as unintentional were recoded with a possible or definitive maltreatment code). However, this study selected a random sample of *all *injury diagnoses across a broad range of ages. A more targeted sample using common maltreatment-related injury codes (such as head injuries, rib fractures etc) within specific age ranges (such as those under age 1), may produce a higher proportion of potential maltreatment-related cases which weren't assigned a maltreatment code. Previous research has identified a range of ICD diagnosis codes beyond the maltreatment-specific ICD codes which could be used to identify samples of cases for further investigation, and a recent systematic review of the literature summarised these studies [15]. Future research should include studies to examine the clinical documentation and reliability of coding of maltreatment for cases with clinical indicators of maltreatment but where no maltreatment codes have been assigned.

It is important to note that the authors have deliberately avoided using the terms 'correct' and 'incorrect' coding or 'accuracy of coding' throughout this article as disagreement in coding between the original coder and the auditor could reflect errors on the part of either the original coder or the auditor. Clinical coding is a complex process and documentation is often not comprehensive, clearly or consistently recorded and hence two coders with the same medical record may assign different codes. Coding is generally performed by multiple coders in a large number of hospitals across Australia, fluctuations between the auditors in their external cause coding are likely to reflect normal variability in the coding process. By referring to the level of agreement or concordance between coders, we are able to estimate the reliability without making assumptions of accuracy on the part of either coder.

## Conclusion

Given the increasing international attention being given to improving the uniformity of reporting of child-maltreatment related injuries and the emphasis on the better utilisation of routinely collected health data, this study provides an estimate of the reliability of maltreatment-specific ICD-10-AM codes. Reliable data is critical if these data are to be used for estimating the incidence and prevalence of child maltreatment resulting in hospitalisation, for describing patterns and estimating risks, for validating data from other sectors (such as police and child safety data), and for providing estimates of the costs of child maltreatment.

## Competing interests

The authors declare that they have no competing interests.

## Authors' contributions

All authors made an important contribution to the development of the ideas and conduct of the research. GW and MC recoded all data and provided expert advice in regards to the comparisons of data; DS and KM assisted in data collection; KM and DS were primarily responsible for developing the first drafts of the manuscript; KM conducted all analysis. All authors have been integrally involved in reviewing and extensively editing the text as it has progressed through several iterations.

## Pre-publication history

The pre-publication history for this paper can be accessed here:

http://www.biomedcentral.com/1471-2458/11/8/prepub
